# Inconclusive Findings: Now You See Them, Now You Don’t!

**DOI:** 10.1289/ehp.1408106

**Published:** 2014-02-01

**Authors:** Christopher J. Portier, Lynn R. Goldman, Bernard D. Goldstein

**Affiliations:** 1 International Agency for Research on Cancer (Senior Visiting Scientist), Lyon, France; 2 George Washington University School of Public Health and Health Services, Washington, DC, USA; 3 Graduate School of Public Health, University of Pittsburgh,Pittsburgh, Pennsylvania, USA; E-mail: cportier@mac.com

The environmental health literature is rife with controversial papers that evoke criticism, support, and, most importantly, a desire to better understand the findings put forth by the authors. A research article by Séralini and colleagues (Séralini et al. 2012), published in the journal *Food and Chemical Toxicology* (*FCT*), is one such article resulting in considerable discourse (Arjó et al. 2013; Barale-Thomas 2013; Grunewald and Bury 2013; Ollivier 2013; Wagner et al. 2013; Sanders et al. 2013; Schorsch 2013; Séralini et al. 2013) and a call for new research (European Commission 2013). This is all part of the scientific process in a modern research environment. However, the retraction of the article by Séralini et al. from *FCT* sets a new precedent in the management of peer-reviewed publications that we believe has serious implications for environmental public health. The retraction announcement by the Editor-in-Chief specifically states, “Ultimately, the results presented (while not incorrect) are inconclusive, and therefore do not reach the threshold of publication for Food and Chemical Toxicology” (FCT 2013). The Editor-in-Chief also was very clear that he “found no evidence of fraud or intentional misrepresentation of the data.”

This article (Séralini et al. 2012) has been controversial from its initial publication. We do not wish to discuss the merits of the authors’ conclusions or their implications for the commercial products in question. Those issues have been debated in the open scientific literature since the publication of the paper, and we agree with many of the critiques. However, the retraction of any paper because it is “inconclusive” has adverse implications on the integrity of the concept of the peer-review process as the critical foundation of unbiased scientific inquiry.

The paper was peer reviewed by scientists on behalf of the *FCT* and published accordingly. Hence, it initially met the threshold for publication. In our opinion, there must be a different threshold for forced retraction of the paper, and we believe that this paper did not reach that threshold. The COPE guidelines for retracting articles (Committee on Publication Ethics 2009) provide four reasons for retraction: scientific misconduct/honest error, prior publication, plagiarism, or unethical research. None of these reasons apply to this particular article, and yet Elsevier, a member of COPE, chose to retract the paper.

The nature of science is such that individual studies are rarely, if ever, conclusive. Numerous published studies have later been found to be deeply flawed through further scientific investigation, as may well be the study by Séralini et al. To our knowledge, there is no precedent for “inconclusive data” being a reason for retraction for Elsevier or other publishers, or elsewhere in the scientific literature. To single out this one study for retraction is almost certainly due to the controversy following its publication. The repercussions of this directed action extend well beyond this single publication and raise several larger scientific questions. Will these data, which could well have been accepted by another journal, now be tainted beyond possibility for inclusion in usual weight-of-evidence reviews of the body of peer-reviewed science? Will the response to new science by interested parties now be focused on dueling attempts to have the paper retracted rather than on performing additional studies to replicate or refute the findings? Does this retraction strengthen the scientific process, or does it confuse scientific discourse with public relations?

Efforts to suppress scientific findings, or the appearance of such, erode the scientific integrity upon which the public trust relies. The retraction by the *FCT* marks a significant and destructive shift in management of the publication of controversial scientific research. Equally troublesome is that this retraction does not really impact how the science will be viewed by scientists, but only how it is viewed by others outside of the scientific community. We feel the decision to retract a published scientific work by an editor, against the desires of the authors, because it is “inconclusive” based on a *post ho*c analysis represents a dangerous erosion of the underpinnings of the peer-review process, and Elsevier should carefully reconsider this decision.
